# Supplementary material for: Seminal fluid compromises visual perception in honeybee queens reducing their survival during additional mating flights
Source: eLife. 2019 Sep 10;8:e45009. doi: 10.7554/eLife.45009 (PMC6739865; doi:10.7554/eLife.45009)
Supplement: Supplementary file 1. — The total number of DEGs identified, the number of these DEGs that were up-regulated, and the number of DEGs that were down-regulated are reported in separate columns for each of the pair-wise comparisons between treatment groups in both RNA-sequencing experiments. [file elife-45009-supp1.docx]

**Supplementary File 1** Summary of results from differential expression analyses performed with DESeq2 (6). The total number of DEGs identified, the number of these DEGs that were up-regulated, and the number of DEGs that were down-regulated are reported in separate columns for each of the pair-wise comparisons between treatment groups in both RNA-sequencing experiments.

| **Experiment** | **Treatment comparison** | **N DEGs** | **Up-regulated** | **Down-regulated** |
| --- | --- | --- | --- | --- |
| 1 | Semen vs Mock insemination | 90 | 68 | 22 |
| 1 | Seminal fluid vs Mock insemination | 264 | 169 | 95 |
| 1 | Seminal fluid vs Semen | 1 | 1 | 0 |
| 2 | Semen vs Hayes saline | 333 | 268 | 65 |
| 2 | Seminal fluid vs Hayes saline | 269 | 150 | 119 |
| 2 | Seminal fluid vs Semen | 802 | 255 | 547 |
