# Supplementary material for: Seminal fluid compromises visual perception in honeybee queens reducing their survival during additional mating flights
Source: eLife. 2019 Sep 10;8:e45009. doi: 10.7554/eLife.45009 (PMC6739865; doi:10.7554/eLife.45009)
Supplement: Supplementary file 9. — df = degrees of freedom, χ²=chi squared statistic. The final model is shown below the table. [file elife-45009-supp9.docx]

| **Supplementary File 9** Results of a linear mixed effects model for flicker response amplitude of compound eyes comparing the measurements taken on the second day post-inseminations for the 9 queens that had exclusively been measured at day 2 with those of the 18 queens that were measured both on day 1 and 2. df = degrees of freedom, χ² = chi-squared statistic. The final model is shown below the table.   \| **response variable** \| **fixed effect** \| **df** \| **χ²** \| ***P* value** \| \| --- \| --- \| --- \| --- \| --- \| \| flicker amplitude \| day2only:frequency \| 6 \| 8.566 \| 0.1995 \| \| final model: amplitude ~ frequency*contrast + treatment + frequency*treatment + day2only*frequency + (1\|animal) + (1\|date) + (1\|chamber) \| \| \| \| \| |
| --- | --- | --- | --- | --- | --- | --- | --- | --- | --- | --- | --- | --- | --- | --- | --- |
