# Supplementary material for: Seminal fluid compromises visual perception in honeybee queens reducing their survival during additional mating flights
Source: eLife. 2019 Sep 10;8:e45009. doi: 10.7554/eLife.45009 (PMC6739865; doi:10.7554/eLife.45009)
Supplement: Supplementary file 10. — Significant effects (p<0.05) are reported in bold. df = degrees of freedom, χ²=chi squared statistic. The final model is shown below the table. [file elife-45009-supp10.docx]

| **Supplementary File 10** Results of a linear mixed effects model for flicker response amplitude of compound eyes after excluding all semen measurements, showing the significance of the fixed effects and their interactions. Significant effects (*P* < 0.05) are reported in bold. df = degrees of freedom, χ² = chi-squared statistic. The final model is shown below the table. | | | | |
| --- | --- | --- | --- | --- |
| **response variable** | **fixed effects** | **df** | **χ²** | ***P* value** |
| flicker amplitude | **intensity** | **12** | **75.908** | **2.47E-11** |
|  | **frequency:contrast** | **12** | **75.908** | **2.47E-11** |
|  | **treatment:day** | **1** | **15.165** | **9.85E-05** |
| final model: amplitude ~ frequency*contrast + intensity + treatment*day + (1\|anim) + (1\|date) + (1\|chamber) | | | | |
