# Supplementary material for: Seminal fluid compromises visual perception in honeybee queens reducing their survival during additional mating flights
Source: eLife. 2019 Sep 10;8:e45009. doi: 10.7554/eLife.45009 (PMC6739865; doi:10.7554/eLife.45009)
Supplement: Supplementary file 11. — Significant effects (p<0.05) are reported in bold. df = degrees of freedom, χ²=chi squared statistic. The final model is shown below the table. [file elife-45009-supp11.docx]

| **Supplementary File 11** Linear mixed effect model for flicker response amplitude of ocelli, showing significant factors and their interactions. Significant effects (*P* < 0.05) are reported in bold. df = degrees of freedom, χ² = chi-squared statistic. The final model is shown below the table. | | | | |
| --- | --- | --- | --- | --- |
| **response variable** | **fixed effects** | **df** | **χ²** | ***P* value** |
| flicker amplitude | **frequency:contrast** | **8** | **25.23** | **0.0014** |
|  | **contrast:treatment** | **4** | **16.53** | **0.0024** |
|  | **treatment:day** | **2** | **8.33** | **0.0155** |
| final model: amplitude ~ frequency*contrast + contrast*treatment + treatment*day + (1\|anim) + (1\|date) + (1\|chamber) | | | | |
|  |  |  |  |  |
