# Supplementary material for: Seminal fluid compromises visual perception in honeybee queens reducing their survival during additional mating flights
Source: eLife. 2019 Sep 10;8:e45009. doi: 10.7554/eLife.45009 (PMC6739865; doi:10.7554/eLife.45009)
Supplement: Supplementary file 13. — Significant effects (p<0.05) are reported in bold. df = degrees of freedom, χ²=chi squared statistic. The final model is shown below the table. [file elife-45009-supp13.docx]

| **Supplementary File 13** Results of a linear mixed effects model for contrast sensitivity of compound eyes, showing the significance of the fixed effects and their interactions. Significant effects (*P* < 0.05) are reported in bold. df = degrees of freedom, χ² = chi-squared statistic. The final model is shown below the table. | | | | |
| --- | --- | --- | --- | --- |
| **response variable** | **fixed effects** | **df** | **χ²** | ***P* value** |
| contrast sensitivity | **frequency:intensity** | **10** | **233.51** | **< 2.2e-16** |
|  | **frequency:treatment** | **10** | **24.052** | **0.008** |
|  | day:treatment | 5 | 11.017 | 0.051 |
| final model: contrast sensitivity ~ frequency*intensity + frequency*treatment + (1\|anim) + (1\|date) + (1\|chamber) | | | | |
|  |  |  |  |  |
