# Supplementary material for: Seminal fluid compromises visual perception in honeybee queens reducing their survival during additional mating flights
Source: eLife. 2019 Sep 10;8:e45009. doi: 10.7554/eLife.45009 (PMC6739865; doi:10.7554/eLife.45009)
Supplement: Supplementary file 14. — Significant effects (p<0.05) are reported in bold. df = degrees of freedom, χ²=chi squared statistic. The final model is shown below the table. [file elife-45009-supp14.docx]

| **Supplementary File 14** Results of a linear mixed effects model for contrast sensitivity of compound eyes after exclusion of semen measurements, showing the significance of the fixed effects and their interactions. Significant effects (*P* < 0.05) are reported in bold. df = degrees of freedom, χ² = chi-squared statistic. The final model is shown below the table. | | | | |
| --- | --- | --- | --- | --- |
| **response variable** | **fixed effects** | **df** | **χ²** | ***P* value** |
| contrast sensitivity | **frequency:intensity** | **2** | **72.737** | **< 2.2e-16** |
|  | frequency:treatment | 2 | 1.718 | 0.424 |
|  | day:treatment | 3 | 0.989 | 0.804 |
| final model: contrast sensitivity ~ frequency*intensity + (1\|anim) + (1\|date) + (1\|chamber) | | | | |
|  |  |  |  |  |
